# Skull base osteosarcoma presenting with cerebrospinal fluid leakage after CyberKnife^®^ treatment: a case report

**DOI:** 10.1186/1752-1947-7-116

**Published:** 2013-04-26

**Authors:** Shoko Merrit Yamada, Yudo Ishii, So Yamada, Yoshiaki Goto, Mineko Murakami, Katsumi Hoya, Akira Matsuno

**Affiliations:** 1 Department of Neurosurgery, Teikyo University Chiba Medical Center, 3426-3 Anesaki, Ichihara-city, Chiba-prefecture 299-0111, Japan; 2 Department of Neurosurgery, Nippon Medical School, 1-1-5 Sendagi, Bunkyo-ku, Tokyo, 113-8603, Japan

**Keywords:** Cerebrospinal fluid, Complication, CyberKnife^®^, Radiation, Skull base, Tumor

## Abstract

**Introduction:**

CyberKnife^®^ radiation is an effective treatment for unresectable skull base tumors because it can deliver a highly conformational dose distribution to the complex shapes of tumor extensions. There have been few reports of severe complications with this treatment. This is the first published case report to our knowledge of cerebrospinal fluid leakage induced by CyberKnife^®^ radiotherapy.

**Case presentation:**

A skull base tumor was identified on magnetic resonance imaging in a 78-year-old Asian woman with a headache in her forehead. An endoscopic transnasal tumor resection was performed; however, the tumor, invading into the cavernous sinuses and optic canal, was not completely removed. During the subtotal resection of the tumor, no cerebrospinal fluid leakage was observed. Osteosarcoma was histologically diagnosed, and CyberKnife^®^ radiation was performed to the residual tumor considering the aggressive feature of the tumor with a molecular immunology Borstel-1 index of 15%. Five months after the treatment, magnetic resonance imaging showed definite tumor shrinkage, and the patient had been living her daily life without any troubles. After another month, the patient was transferred to our clinic because of coma with high fever, and computed tomography demonstrated severe pneumocephalus. Rhinorrhea was definitely identified on admission; therefore, emergency repair of the cerebrospinal fluid leakage was performed using an endoscope. Dural defects at the bottom of the sella turcica were identified under careful endoscopic observation and fat tissue was patched to the dural defects. Follow-up computed tomography proved complete disappearance of air from the cisterns 2 weeks after the surgery, and the patient was discharged from our hospital without any neurological deficits.

**Conclusion:**

CyberKnife^®^ radiation is one of the effective treatments for skull base tumors; however, the risk of cerebrospinal fluid leakage should be considered when tumor invasion to the dura mater is suspected. Emergency surgical treatment is required when cerebrospinal fluid leakage is induced by the radiotherapy because the leakage is not expected to be healed by palliative treatments.

## Introduction

Skull base tumors are frequently aggressive and total resection of the tumors is very difficult because of neurovascular anatomical features. Irradiation is well indicated for unresectable skull base tumors. Fractionated CyberKnife^®^ radiation delivers a highly conformational dose distribution to the complex shapes of tumor extensions into the skull base [1]. It is clear that many patients with skull base tumors have benefited from this radiosurgery [2-4]. Here we present a case of skull base osteosarcoma which, remarkably, was shrunk by CyberKnife^®^ radiation, but cerebrospinal fluid (CSF) leakage occurred due to the tumor shrinkage.

## Case presentation

**Figure 1:**
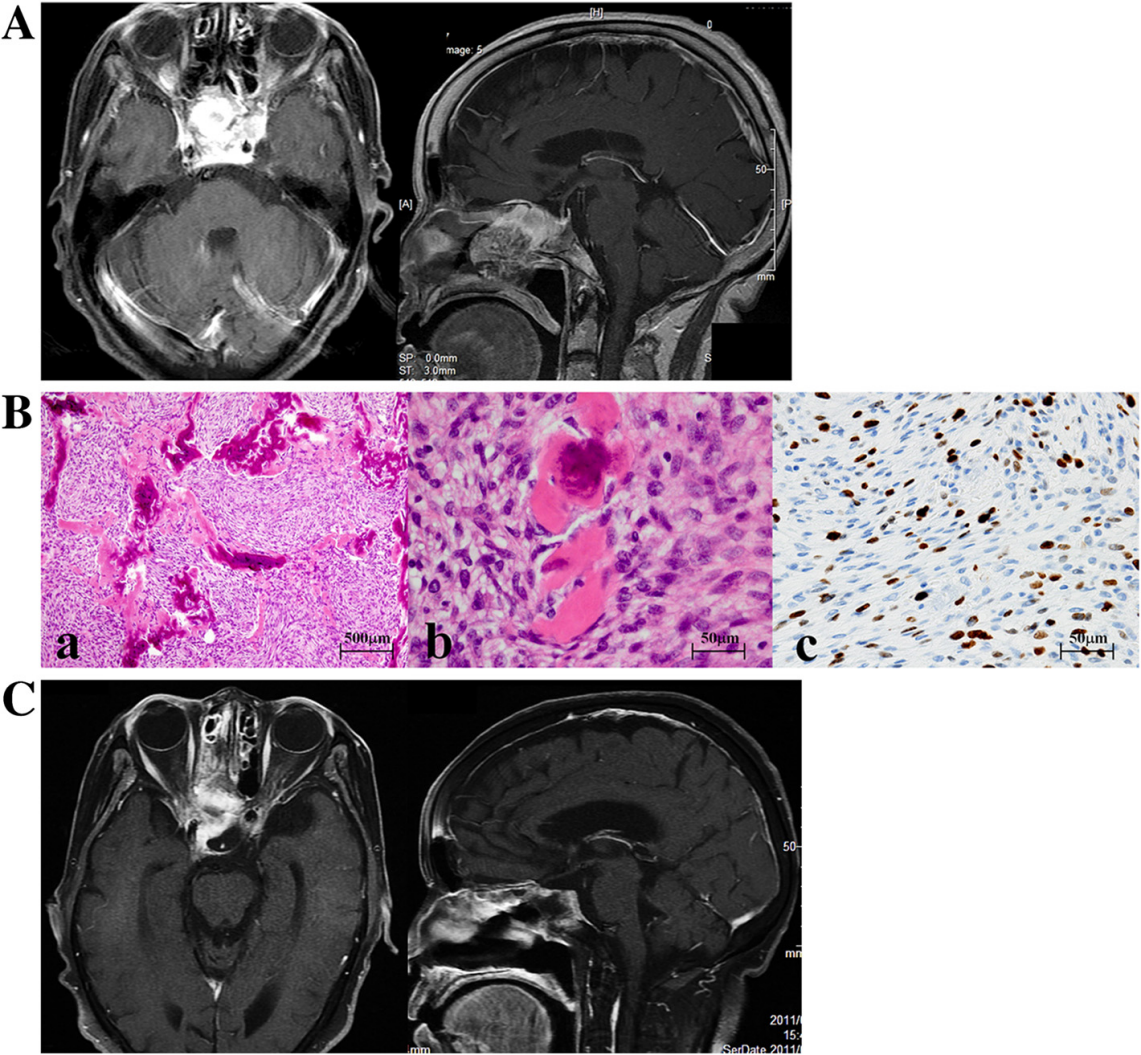
**A. Enhanced T1-weighted magnetic resonance imaging. **Enhanced mass occupies sphenoid sinus entirely. The tumor has invaded the bilateral cavernous sinuses surrounding internal carotid arteries, but pituitary gland and stalk seem to be totally intact (sagittal view). **B. Histology of the tumor. **Hematoxylin and eosin staining shows high cellularity in the tumor tissue (**a**), and osseous components mixed in with the tumor (**b**) suggesting osteosarcoma. Molecular immunology Borstel-1 (MIB-1) index was high, 15% (c). **C. Postoperative magnetic resonance imaging. **Magnetic resonance imaging showed a reduction of the tumor volume in the sphenoid and ethmoid sinuses, but residual tumor definitely exists in the sinuses with strong enhancement.

**Figure 2:**
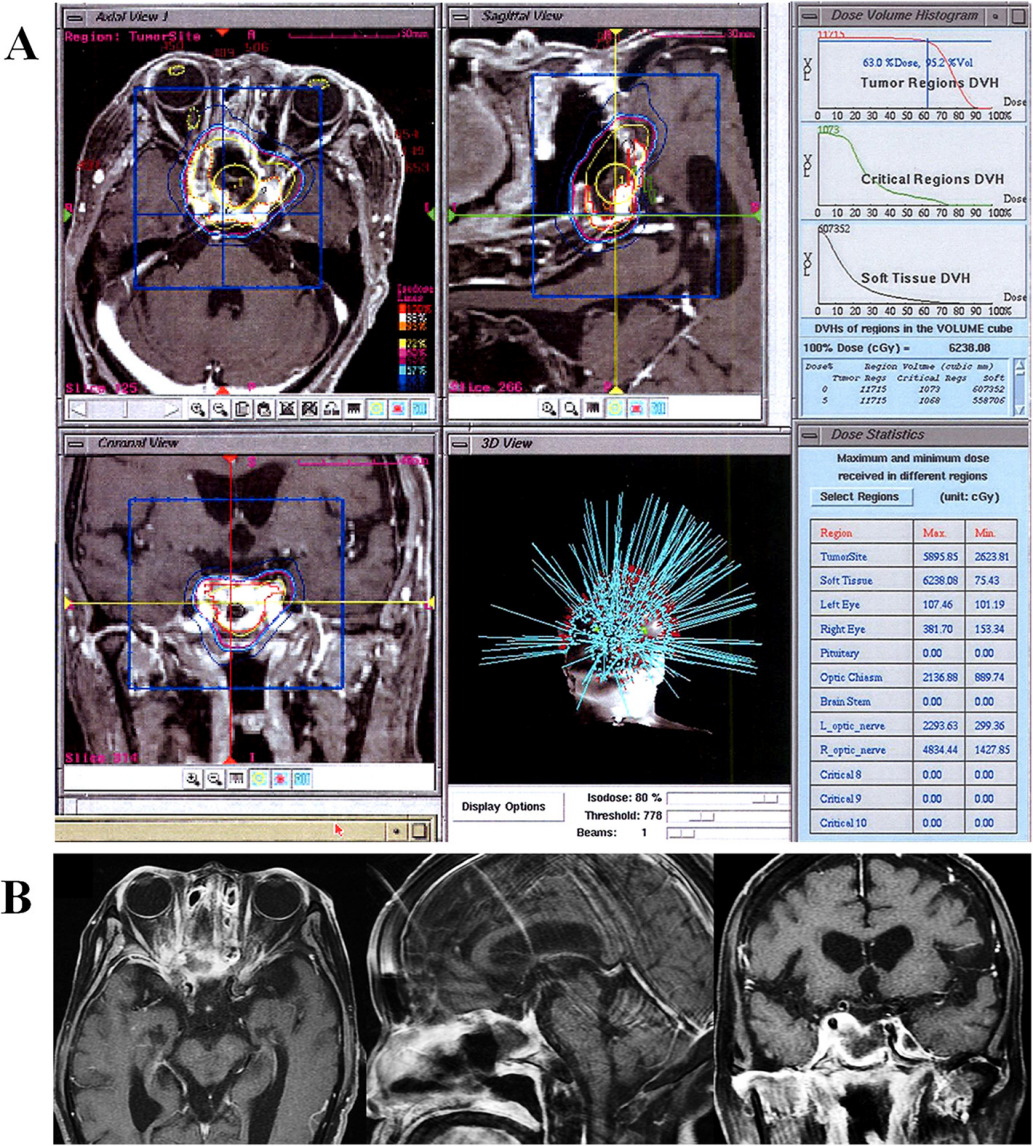
**A. Planning of CyberKnife^® ^radiation. **The planned radiation was applied in five fractions, mainly to the residual tumor in the sphenoid and ethmoid sinuses including the optic canals. DVH: dose volume histogram. **B. Follow-up magnetic resonance imaging performed 5 months after CyberKnife^® ^treatment. **The residual tumor exactly shrinks after CyberKnife^® ^radiation. Of note, the size of enhanced lesions in the cavernous sinuses has decreased remarkably.

**Figure 3:**
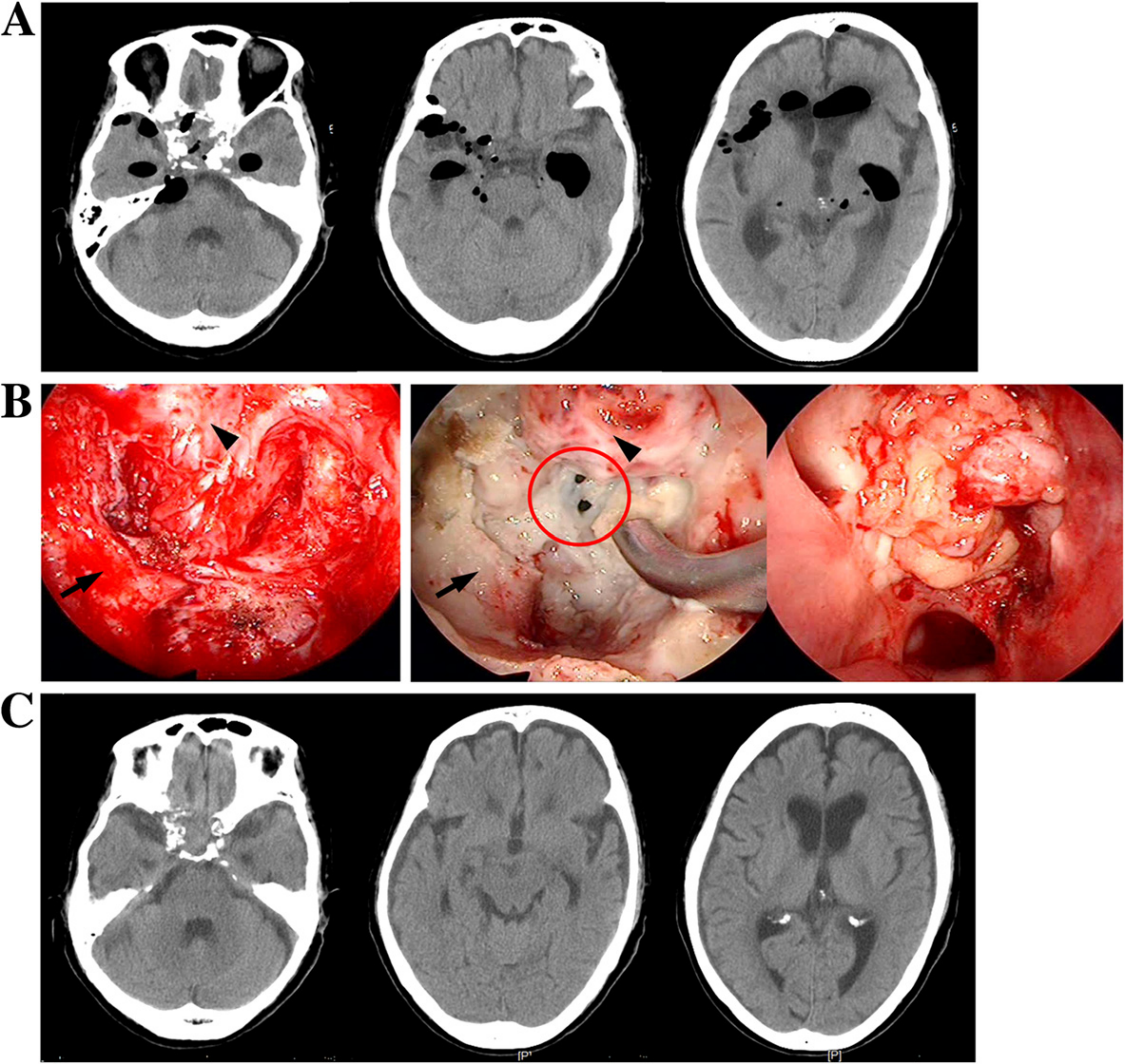
**A. Computed tomography 6 months after CyberKnife^® ^treatment. **Multiple extremely low density areas are shown in subarachnoid spaces and lateral ventricles, suggesting pneumocephalus. **B. Endoscopic repair of cerebrospinal fluid (CSF) leakage.** Left: The endoscopic appearance of subtotal tumor resection in the first surgery. Center: Endoscopic observation of the residual tumor 6 months after CyberKnife^®^ treatment. All irradiated tissue is hypovascular and whitish, and two small holes are identified in the dura mater at the bottom of sella turcica (red circle). Right: Fat tissue was used as a patch and fixed in place with fibrin glue. Black arrows show the lateral bone of the floor of the sella turcica and black arrowheads indicate residual tumor. **C. Computed tomography after repair of CSF leakage. **Air had completely disappeared 2 weeks after the endoscopic repair of CSF leakage.

A 78-year-old Asian woman visited our clinic complaining of a headache in her forehead. Magnetic resonance imaging (MRI) demonstrated an enhanced mass in the sphenoid and ethmoid sinuses with invasion into cavernous sinuses (Figure 1A). The skull base tumor was considered a cause of her headache, and endoscopic transnasal tumor resection was performed. The bone in the floor of the sella turcica was destroyed by the tumor, which impinged upon a small area of dura mater in the bottom of the sella. A large part of the tumor extended into the extradural space; however, the tumor could not be separated from the dura completely because of its strong adhesion and invasion to the dura. To avoid surgical damage to the internal carotid artery, the tumor invading the cavernous sinuses was not aggressively removed. Osteosarcoma was histologically diagnosed with the following characteristics: hematoxylin and eosin staining showed high cellularity of the tumor with pleomorphism (Figure 1B-a), osseous tissue depositions were identified (Figure 1B-b), and molecular immunology Borstel-1 (MIB-1) index was 15% (Figure 1B-c). Definite residual tumor was recognized in the sphenoid and ethmoid sinuses in the postoperative MRI (Figure 1C). Considering both the effectiveness of radiation therapy for sarcomas [5] and the shape of the residual tumor, CyberKnife^®^ radiation was applied 1 month after the surgery. The treatment was performed on the basis of the MRI planning (Figure 2A) with the following parameters: target volume, 11716mm^3^; collimator, 20mm; number of fractions, five; marginal dose, 39.38Gy; and site dose, 55.69Gy. Five months after CyberKnife^®^ treatment, the patient was living a normal life in her home without demonstrating any neurological deficits, and follow-up MRI showed shrinking of the residual tumor (Figure 2B). However, 1 month later, the patient was transferred to our hospital because of coma with high fever. Computed tomography (CT) showed multiple air pockets in the basal cisterns, the subarachnoid space, and lateral ventricles (Figure 3A); rhinorrhea was identified after admission. Emergency repair of CSF leakage was performed by an endoscopic transnasal approach. Compared with the endoscopic appearance of the residual tumor in the first surgery (Figure 3B left), the irradiated tumor tissue was whitish with a smooth surface suggesting hypovascularity (Figure 3B center). Under careful endoscopic observation, efflux of CSF synchronizing with pulsation was identified from two small dural defects where the osteosarcoma had invaded (Figure 3B red circle). Fat tissue was widely patched over the defects and fixed in place with fibrin glue (Figure 3B right). No rhinorrhea was recognized after the surgery, and follow-up CT showed complete disappearance of air from the cisterns 2 weeks after the surgery (Figure 3C). The patient fully recovered her condition 1 month after the surgery.

## Discussion

In skull base tumors, the portions where surgical resection is difficult are also areas where radiation therapy is challenging, such as the cavernous sinus or optic canal, which include cranial nerves and arteries. Cranial nerve palsies and internal carotid artery stenosis caused by external radiation have been reported [6,7]. To preserve the function of optic nerves, fractionated CyberKnife^®^ radiation is safer and more effective than conventional radiation or gamma knife treatment [6]. In our case, CyberKnife^®^ treatment shrank the osteosarcoma without causing visual deterioration. However, critical CSF leakage occurred. As shown in Figure 3B left, endoscopic observation revealed thick residual tumor tissue invading the dura mater at the bottom of the sella before CyberKnife^®^ irradiation, and the residual tumor protected the patient from CSF leakage. After the treatment, the irradiated tissue became hypovascular and necrotic change was identified in the residual tumor progressing to the dural defects. As another cause of the CSF leakage, it might be possible that the authors injured dura mater at the sella during the removal of the tumor because the border between the tumor and the dura was not observable at some points. And this pre-existing iatrogenic dural defect caused CSF leakage when the dura shrank in CyberKnife^®^ therapy. To the best of our knowledge this is the first reported case of CSF leakage caused by CyberKnife^®^ therapy, although a few cases of post-radiation CSF leakage were reported in gamma-knife treatment for skull base tumors [8-10]. There is no doubt that CyberKnife^®^ treatment is very effective for skull base tumors; [2-4] however, the risk of radiation-induced CSF leakage should be considered when tumor invasion of the dura is suspected. Emergency surgical repair is required if CSF leakage is identified to avoid severe meningitis, because CSF leakage cannot be cured by palliative treatments. When a skull base tumor extends widely, it might be difficult to identify the point of CSF leakage induced by radiotherapy. In such a case, extended subcranial approach, which is usually performed for repairing rhinorrhea caused by anterior skull base fracture [11], should be considered instead of endoscopic transnasal surgery.

## Conclusion

The risk of CSF leakage caused by CyberKnife^®^ radiation should be considered when tumor invasion to the dura mater is recognized. Emergency surgical treatment is required when CSF leakage is identified because the leakage is not healed with conservative treatment.

## Consent

Written informed consent was obtained from the patient for publication of this manuscript and any accompanying images. A copy of the written consent is available for review by the Editor-in-Chief of this journal.

## Abbreviations

CSF: Cerebrospinal fluid; CT: Computed tomography; MRI: Magnetic resonance imaging.

## Competing interests

The authors declare that they have no competing interests.

## Authors’ contributions

SMY was a major contributor in writing the manuscript. YI, SMY, and SY performed surgery and analyzed the data. YI and YG accomplished emergency repair of CSF leakage. MM, KH, and AM revised the manuscript with important intellectual content. All authors read and approved the final manuscript.

## References

[B1] Chang SD Main W Martin DP Gibbs IC Heilbrun MP An analysis of the accuracy of the CyberKnife: a robotic frameless stereotactic radiosurgical system Neurosurgery 2003 52 140 146 discussion 146–147 12493111 10.1097/00006123-200301000-00018

[B2] Tuniz F Soltys SG Choi CY Chang SD Gibbs IC Fischbein NJ Adler JR Jr Multisession cyberknife stereotactic radiosurgery of large, benign cranial base tumors: preliminary study Neurosurgery 2009 65 898 907 discussion 907 10.1227/01.NEU.0000359316.34041.A8 19834402

[B3] Bianchi LC Marchetti M Brait L Bergantin A Milanesi I Broggi G Fariselli L Paragangliomas of head and neck: a treatment option with CyberKnife radiosurgery Neurol Sci 2009 30 479 485 10.1007/s10072-009-0138-3 19774334

[B4] Collins SP Coppa ND Zhang Y Collins BT McRae DA Jean WC CyberKnife radiosurgery in the treatment of complex skull base tumors: analysis of treatment planning parameters Radiat Oncol 2006 1 46 10.1186/1748-717X-1-46 17173702 PMC1764417

[B5] DeLaney TF Trofimov AV Engelsman M Suit HD Advanced-technology radiation therapy in the management of bone and soft tissue sarcomas Cancer Control 2005 12 27 35 Review 15668650 10.1177/107327480501200104

[B6] Pham CJ Chang SD Gibbs IC Jones P Heilbrun MP Adler JR Jr Preliminary visual field preservation after staged CyberKnife radiosurgery for perioptic lesions Neurosurgery 2004 54 799 810 discussion 810–812 10.1227/01.NEU.0000114261.18723.6A 15046645

[B7] Houdart E Mounayer C Chapot R Saint-Maurice JP Merland JJ Carotid stenting for radiation-induced stenoses: a report of 7 cases Stroke 2001 32 118 121 10.1161/01.STR.32.1.118 11136925

[B8] Ogawa Y Tominaga T Delayed cerebrospinal fluid leakage 10 years after transsphenoidal surgery and gamma knife surgery – case report –Neurol Med Chir (Tokyo) 2007 47 483 485 10.2176/nmc.47.483 17965568

[B9] Kim CH Chung SK Dhong HJ Lee JI Cerebrospinal fluid leakage after gamma knife radiosurgery for skull base metastasis from renal cell carcinoma: a case report Laryngoscope 2008 118 1925 1927 10.1097/MLG.0b013e3181820171 18797420

[B10] Hongmei Y Zhe W Jing W Daokui W Peicheng C Yongjie L Delayed cerebrospinal fluid rhinorrhea after gamma knife surgery in a patient with a growth hormone-secreting adenoma J Clin Neurosci 2012 19 900 902 10.1016/j.jocn.2011.09.016 22349430

[B11] Schaller B Subcranial approach in the surgical treatment of anterior skull base trauma Acta Neurochir (Wien) 2005 147 355 366 discussion 366 10.1007/s00701-005-0487-5 15726278

